# Genome-wide screen identifies a novel prognostic signature for breast cancer survival

**DOI:** 10.18632/oncotarget.14776

**Published:** 2017-01-21

**Authors:** Xuan Y Mao, Matthew J Lee, Jeffrey Zhu, Carissa Zhu, Sindy M Law, Antoine M Snijders

**Affiliations:** ^1^ Biological Systems and Engineering Division, Lawrence Berkeley National Laboratory, Berkeley, California, USA; ^2^ Department of Psychiatry, Weill Institute for Neurosciences, University of California San Francisco, San Francisco, California, USA

**Keywords:** breast cancer, prognostic score, relapse-free survival, gene biomarkers

## Abstract

Large genomic datasets in combination with clinical data can be used as an unbiased tool to identify genes important in patient survival and discover potential therapeutic targets. We used a genome-wide screen to identify 587 genes significantly and robustly deregulated across four independent breast cancer (BC) datasets compared to normal breast tissue. Gene expression of 381 genes was significantly associated with relapse-free survival (RFS) in BC patients. We used a gene co-expression network approach to visualize the genetic architecture in normal breast and BCs. In normal breast tissue, co-expression cliques were identified enriched for cell cycle, gene transcription, cell adhesion, cytoskeletal organization and metabolism. In contrast, in BC, only two major co-expression cliques were identified enriched for cell cycle-related processes or blood vessel development, cell adhesion and mammary gland development processes. Interestingly, gene expression levels of 7 genes were found to be negatively correlated with many cell cycle related genes, highlighting these genes as potential tumor suppressors and novel therapeutic targets. A forward-conditional Cox regression analysis was used to identify a 12-gene signature associated with RFS. A prognostic scoring system was created based on the 12-gene signature. This scoring system robustly predicted BC patient RFS in 60 sampling test sets and was further validated in TCGA and METABRIC BC data. Our integrated study identified a 12-gene prognostic signature that could guide adjuvant therapy for BC patients and includes novel potential molecular targets for therapy.

## INTRODUCTION

Breast cancer (BC) is the leading female malignancy and the second leading cause of cancer deaths in U.S. women, with tumor metastasis being the underlying cause in most of these breast cancer related deaths [1, 2]. Breast carcinogenesis is a multi-step process in which epithelial cells accumulate genetic alterations, which in a permissive tissue microenvironment progress towards malignancy and may then metastasize to distant organs. Advances in imaging technologies and heightened public awareness of breast cancer have resulted in an increase in the diagnosis of early-stage breast cancer [3–5]. Furthermore, adjuvant systemic therapy has reduced the risk of recurrence and improved overall survival from BC [6]. However, not all patients who receive adjuvant therapy benefit from it and could have been spared the treatment-associated toxicity. Prognostic factors are critical to distinguish patients with poor prognoses, who would benefit from adjuvant therapy, from patients with good prognoses, who may not benefit sufficiently from adjuvant therapy to outweigh the risks associated with treatment [7].

Traditional prognostic factors currently used to guide the use of systemic therapy and predict outcome include tumor size, lymph node involvement, histological grade, age, race, estrogen receptor (ER), progesterone receptor (PR) and epidermal growth factor receptor (HER2) status [8]. However, a critical problem with BC is the difference in clinical outcome among patients with the same disease. This heterogeneous clinical outcome is manifested by differences in disease susceptibility, progression, treatment response, and relapse, even among individuals with the same apparent histopathological disease. These differences seem to be in part controlled by so-called tumor modifier genes, multiple low-penetrance susceptibility genes that interact with each other and their environment to contribute to the disease process.

Clinical patient survival data, along with genomic datasets can be used to identify genes important in patient survival. Recently, a large gene expression database across normal human tissues became available and which can be used to identify the biological mechanisms underlying different diseases and identify potential novel therapeutic targets [9, 10]. We combined independent BC databases to identify a gene expression signature of differentially expressed genes. Using gene co-expression network analyses, we investigated the genetic architecture of this signature in normal breast tissue. We subsequently identified and validated a 12-gene signature that predicts BC survival.

## RESULTS

### Meta-analysis identified a 587-gene signature frequently deregulated in human breast cancer

**Figure 1:**
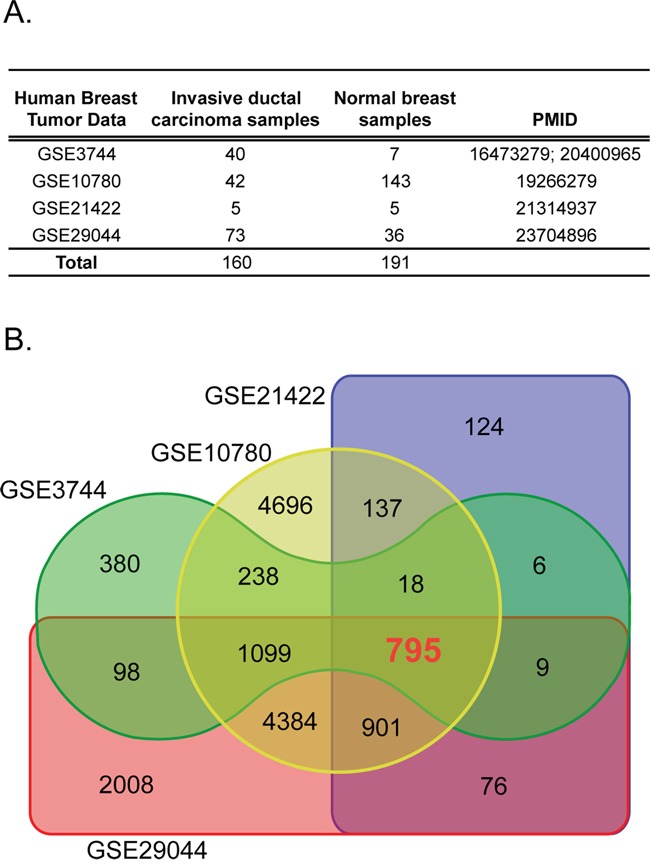
The human breast tissue data sets used in this study **A**. Four independent gene transcript data sets containing invasive ductal carcinoma and normal breast tissue samples were used. **B**. Differential expression of tumor versus normal using a fold-change cut-off of 1.5 and adjusted p-value 0.01 identified the 795 common probe ID set.

**Figure 2:**
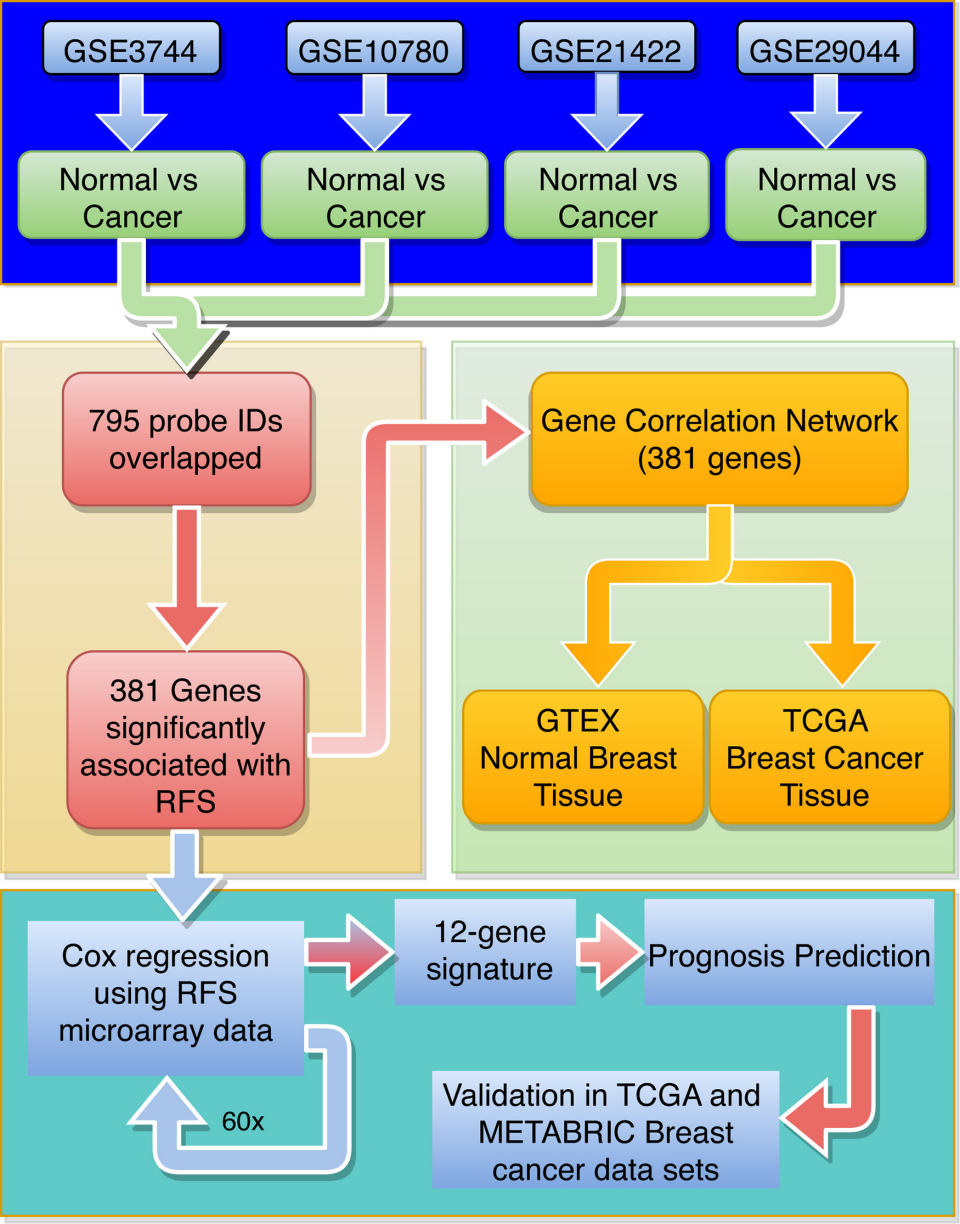
Flow diagram for identifying and validating a prognostic biomarker panel for breast cancer The 795 robustly deregulated probe IDs were identified using 4 breast tumor microarray data sets (blue). To identify individual genes associated with relapse-free survival (RFS), Kaplan Meier survival analysis was run on the overlapping IDs (yellow). A gene expression correlation network approach was used to identify cliques of functionally related genes (green). Cox regression was run on 60 random tumor samples for 381 genes significantly associated with RFS (turquoise) to generate the 12-gene signature. The 12-gene signature was used to generate a prognosis scoring system, which was validated using the TCGA and METABRIC BC data sets.

We conducted a meta-analysis of genes consistently deregulated in human BCs. We collected gene transcript data from normal and tumor breast tissues represented by four independent gene expression data sets totaling 160 invasive ductal carcinomas and 191 normal breast tissues (Figure 1A) [11–15]. The significant differential expression of genes was assessed by a fold change cutoff of 1.5 and adjusted p-value<0.01 (Supplementary Table 1). This resulted in a gene signature of 795 probe IDs (590 down-regulated and 205 up-regulated) represented by 587 unique genes, for which the direction of expression was consistent across all four datasets (Figure 1B and Figure 2; Supplementary Table 2).

### 381 genes significantly associated with relapse-free survival in breast cancer patients

**Figure 3:**
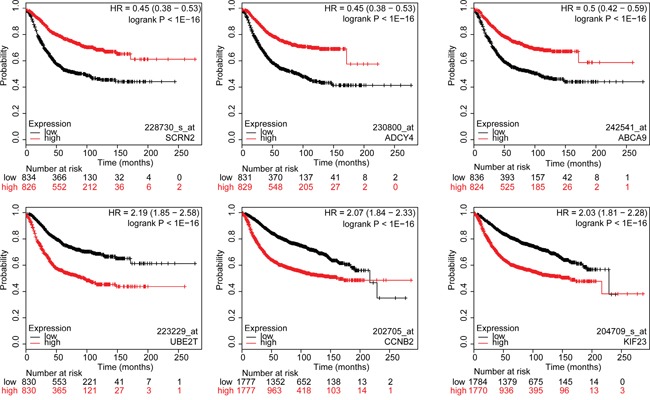
Kaplan-Meier survival curves for breast cancer patients according to tumor expression of genes with highest and lowest hazard ratios The breast cancer patient cohort was divided into two equal groups based on median expression for each gene and compared by a Kaplan-Meier survival analysis. The estimate of the hazard ratio (HR) and log-rank p-value of the curve comparison between the groups is shown. Top three genes with the lowest HR values (top row): SCRN2, ADCY4 and ABCA9. Top three genes with the highest HR values (bottom row): UBE2T, CCNB2 and KIF23. Low and high risks indicated in black and red, respectively.

To investigate whether any of the 587 common deregulated genes were associated with relapse-free survival (RFS), we evaluated the prognostic value for each individual gene in a large public clinical microarray database using the Kaplan-Meier plotter (http://kmplot.com/) (Figure 2) [16]. The BC patient cohort was divided into two equal groups based on median expression for each gene and compared by a Kaplan-Meier survival analysis. In addition, the hazard ratio with a 95% confidence interval and logrank p-value was calculated to evaluate the prognostic significance of each gene for RFS. This analysis identified 381 genes significantly associated with RFS (p-value<6.3E-05; Figure 3, Table 1 and Supplementary Table 3); 249 genes had a hazard ratio < 1 (higher gene expression associated with good prognosis) and 133 genes had a hazard ratio > 1 (higher gene expression associated with poor prognosis).

**Table 1:** Twelve-gene prognostic gene signature

Gene symbol	Gene name	Affymetrix ID	Hazard Ratio	p-value
EPS15	Epidermal growth factor receptor substrate 15	217886_at	0.73	9.30E-08
MELK	Maternal Embryonic Leucine Zipper Kinase	204825_at	1.89	1.00E-16
NUF2	NDC80 Kinetochore Complex Component	223381_at	1.63	2.30E-09
RNASEH2A	Ribonuclease H2 Subunit A	203022_at	1.56	1.90E-14
S100P	S100 Calcium Binding Protein P	204351_at	1.45	2.50E-10
THYN1	Thymocyte Nuclear Protein 1	218491_s_at	0.76	2.70E-06
TIMM17A	Translocase Of Inner Mitochondrial Membrane 17 Homolog A	201821_s_at	1.55	3.70E-14
TSC1	Tuberous Sclerosis 1	209390_at	0.74	4.00E-07
USP47	Ubiquitin Specific Peptidase 47	223119_s_at	0.65	2.40E-07
ZBTB16	Zinc finger and BTB domain containing 16	205883_at	0.6	1.00E-16
PLPP1	Phospholipid Phosphatase 1	209147_s_at	0.77	4.10E-06
PLEKHH2	Pleckstrin Homology, MyTH4 And FERM Domain Containing H2	227148_at	0.59	1.70E-10

### Genes that predict prognosis are enriched for microenvironment- and cell cycle-related biological processes

To reveal the biological functions enriched in the 381-gene set associated with RFS, we performed Gene Ontology analysis separately on the 249 genes that exhibited a HR<1 and 133 genes with HR>1. The 249-gene signature (HR<1) was significantly enriched for tissue microenvironment related processes including cell adhesion (adj. p-value=6E-04), cell migration (adj. p-value=2.74E-05), wound healing (adj. p-value=3.1E-03), and vasculature development (adj. p-value=4.13E-05) (Supplementary Table 4). On the other hand, the 133-gene signature (HR>1) was strongly enriched for cell cycle related processes (adj. p-value=5.33E-51) (Supplementary Table 4). This strong dichotomy between RFS genes with HR<1 - associated with tumor processes enriched for tissue microenvironment-related biological functions (e.g. vasculature, wound healing, cell migration) - and RFS genes with HR>1 - almost exclusively associated with cell cycle related processes - prompted us to further investigate the genetic architecture of these genes in normal breast tissues and BCs.

### Gene co-expression network analysis visualizes the genetic architecture of RFS associated genes in normal breast and breast cancer

**Figure 4:**
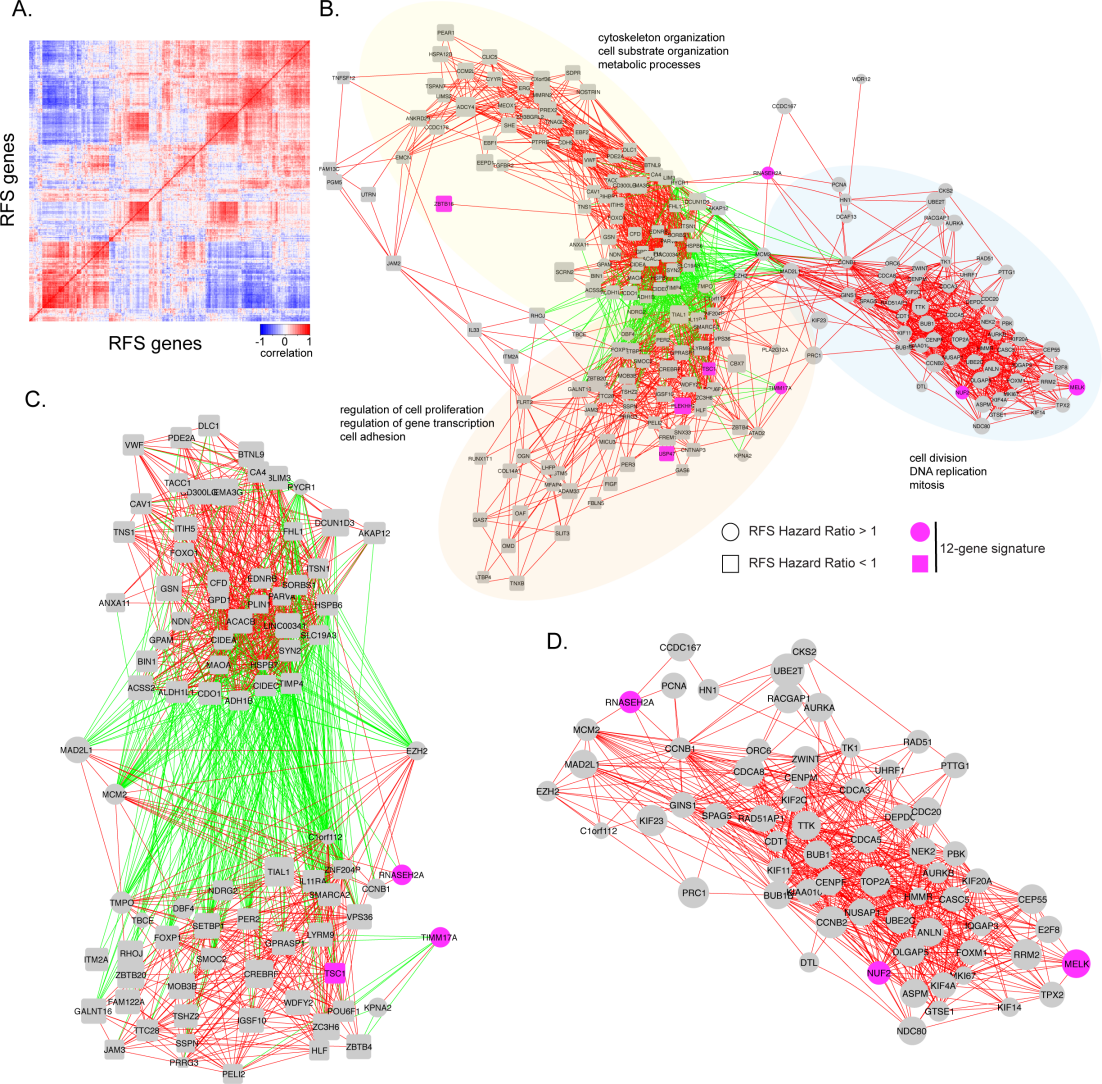
Visual representation of correlations in gene expression in normal human breast tissue samples **A**. The heat map shows the correlation in gene expression between normal breast tissue samples obtained from GTEX. Positive correlations are indicated in red, while negative correlations are indicated in blue. **B**. Gene expression correlation network of RFS significant genes in normal breast tissue samples. Individual genes are indicated as nodes. Red edges indicate a positive correlation in gene expression (r ≥ 0.6) between two genes. Green edges indicate a negative correlation in gene expression between two genes (r ≤ -0.6). Labels indicate significant biological enrichment (adjusted p-value<0.05). Pink colored genes are present in the 12-gene prognostic signature. Three major functional cliques were separated based on gene-ontology. Clique 1 (yellow): cytoskeleton organization, cell substrate organization, and metabolic processes. Clique 2 (orange): regulation of cell proliferation, regulation of gene transcription, and cell adhesion. Clique 3 (blue): ell division, DNA replication, and mitosis. Genes with hazard ratio for RFS >1 are indicated as circles and those with HR<1 as squares. **C**. Enlargement of negative correlations and the genes associated with them. **D**. Enlarged cell division, DNA replication, and mitosis clique.

Since gene sets that are correlated in expression across tissue samples often share a common function, co-expression network analysis has been used to identify clusters of genes with common biological functionality important in normal or tumor tissues. We used data obtained from the GTEX database of 214 normal human breast tissues and the TCGA database of 1100 BC samples to reveal the genetic architecture of RFS associated genes in normal and tumor breast tissue (Figure 2). We first calculated correlation coefficients of 381 genes associated with RFS across 214 normal human breast tissues and 1100 breast cancer samples (Figure 4A). We then constructed a gene expression correlation network where nodes represented individual gene and edges connecting genes represented a correlation in their expression (Figure 4B, R≥|0.6|; p-value<8E-08). In normal breast tissue, three main co-expression cliques were identified (Figure 4B). One clique was highly enriched for genes involved in cell cycle and mitosis, and whose genes all had a hazard ratio for RFS > 1 (Figure 4B, 4D). The remaining two co-expression cliques contained predominantly genes with a hazard ratio for RFS <1. One clique was enriched for genes involved in transcriptional regulation and cell adhesion, while the other clique was generally involved in cytoskeleton organization and metabolic processes. Interestingly, while expression levels of genes within each clique were predominantly positively correlated, expression levels of genes between these two cliques were negatively correlated (Figure 4C). The cell cycle clique is connected to these two cliques through EZH2, MCM2 and MAD2L1.

**Figure 5:**
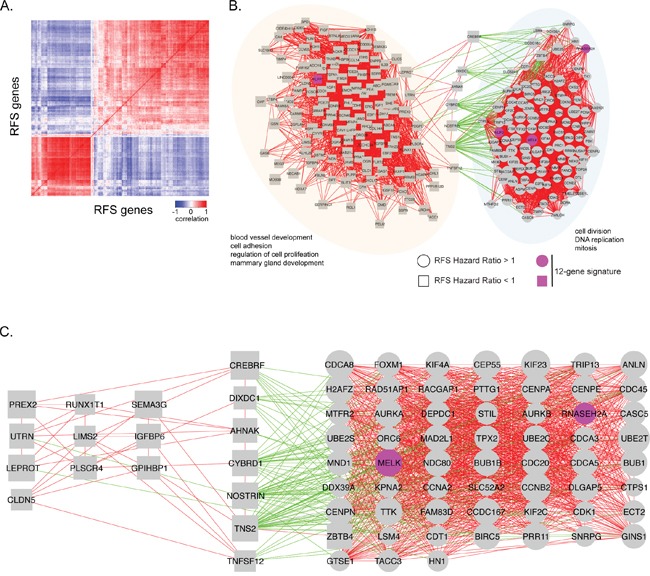
Visual representation of correlations in gene expression in breast cancer samples **A**. The heat map shows the correlation in gene expression between breast cancer samples obtained from TCGA. Positive correlations are indicated in red, while negative correlations are indicated in blue. **B**. Gene expression correlation network of RFS significant genes in breast cancer samples. Individual genes are indicated as nodes. Red edges indicate a positive correlation in gene expression (r ≥ 0.6) between two genes. Green edges indicate a negative correlation in gene expression between two genes (r ≤ -0.6). Labels indicate significant biological enrichment (adjusted p-value<0.05). Pink colored genes are present in the 12-gene prognostic signature. Two major functional cliques were separated based on gene-ontology. Clique 1 (orange): blood vessel development, cell adhesion, regulation of cell proliferation and mammary gland development. Clique 2 (blue): cell division, DNA replication, and mitosis. Genes with hazard ratio for RFS >1 are indicated as circles and those with HR<1 as squares. **C**. Correlation network with negatively correlated genes and its association with cell division, DNA replication, and mitosis genes, as well as some blood vessel development, cell adhesion, regulation of cell proliferation, and mammary gland development genes.

A similar co-expression correlation analysis using TCGA data revealed two main co-expression cliques (Figure 5A, 5B). Similar to normal breast tissue, one clique was highly enriched for genes involved in cell cycle and mitosis, all of which had a hazard ratio for RFS > 1 (Figure 5B). The remaining clique contained genes with a hazard ratio for RFS <1 and was enriched for blood vessel development, cell adhesion and mammary gland development. These two co-expression cliques were negatively correlated through 7 genes: CREBRF, DIXDC1, AHNAK, CYBRD1, NOSTRIN, TNS2 and TNFSF12 (Figure 5C). Given the negative correlation with cell cycle related genes, these 7 genes could mediate negative regulation of cell growth and are potential therapeutic targets.

### A 12-gene prognostic signature predicts breast cancer patient survival

**Figure 6:**
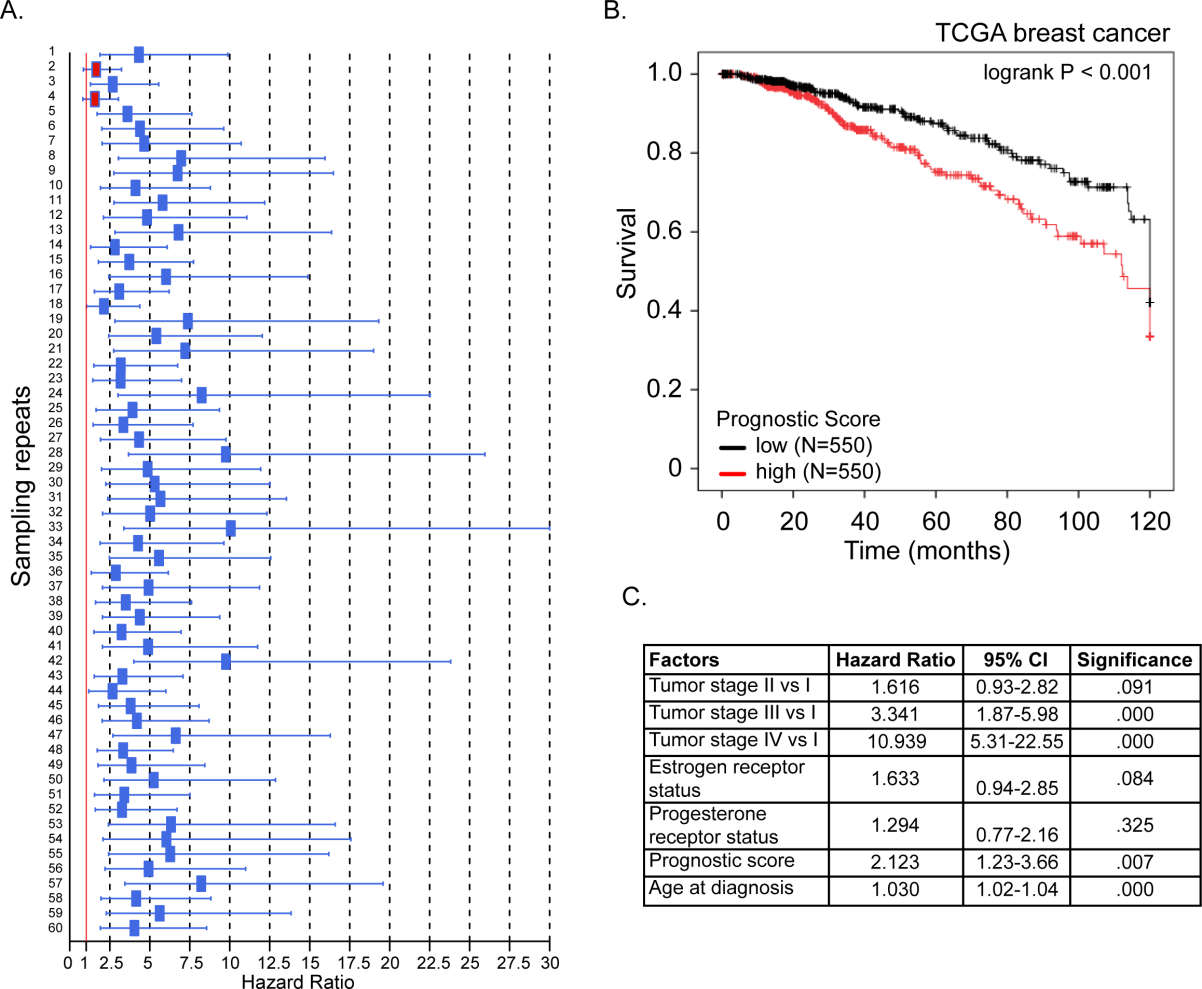
A 12-gene signature predicts breast cancer patient prognosis **A**. For each of 60 test sets the hazard ratio and the 95% confidence interval was calculated using a Cox model based on the prognostic score with groups as covariates, and subsequently plotted in a forest-plot diagram. The red line indicates a HR value of 1, or the null hypothesis. The two red boxes indicate the insignificant trials (confidence interval included HR value of 1) **B**. Kaplan-Meier overall survival curve for breast cancer patients according to prognostic score using the 12-gene signature. The BC patient cohort was divided into two equal groups based on the prognostic score. The log-rank p-value of the curve comparison between the groups is shown. **C**. The hazard ratio and the 95% confidence interval was calculated using a Cox model based on tumor stage (I-IV), estrogen receptor and progesterone receptor status, age at diagnosis and prognostic score as covariates.

Using the 381-gene set associated with RFS we developed a gene signature that accurately predicts BC patient survival (Figure 2). We created 60 training sets through randomly selecting 300 patients each time from the BC gene expression dataset GSE6532, which has RFS information of 393 patients. The residual 93 patients from all 60 training sets formed the 60 test sets. We then performed Cox regression analysis on all 60 training sets to simultaneously assess the importance of the genes within the 381-gene in the RFS. The genes that recurred in at least half of the training sets were included in our final 12-gene signature: EPS15, MELK, NUF2, PLEKHH2, PLPP1, RNASEH2A, S100P, THYN1, TIMM17A, TSC1, USP47, ZBTB16 (Table 1). The average beta-value (Cox regression coefficient) of each of the 12 genes was calculated and used as a weighting factor for the expression value of each gene. A prognostic score was estimated for each patient: gene expression values were multiplied by their respective beta-value and the prognostic score was determined as the sum of resulting weighted gene expression values. The patients were ranked by their prognostic score, divided into two equal sized cohorts based on the median score, and Kaplan-Meier analysis was performed to determine differences in RFS between two cohorts. Using the mean beta values developed in the training set, prognostic scores were calculated for all patients in the 60 test sets. Patients were again ranked on their prognostic score and divided into two cohorts based on the average prognostic-score cut-point in the 60 training sets. Kaplan-Meier analysis was performed and a log-rank test was used to determine if there was a significant difference in RFS between two cohorts. The hazard ratio was calculated for each of the 60 test sets. In only 2 out of 60 (3.3%) test sets, the hazard ratio confidence interval crossed “1” (Figure 6A).

### Validation of 12-gene prognostic signature

We then tested our 12-gene prognostic signature in an independent set of 1100 BC patients obtained from the TCGA database. Prognostic scores for all 1100 patients were calculated and patients were ranked based on their score and divided into two equal sized cohorts. Kaplan-Meier analysis revealed a significant difference between the two patient cohorts. Patients with a high prognostic score had a significantly shorter overall survival compared to patients with a low prognostic score (Figure 6B; p<0.001). To determine if our prognostic score was independent of age at diagnosis, tumor stage, estrogen- and progesterone-receptor status, we ran multivariate Cox regression force-entry with these factors including the prognostics scores as covariates. We found that prognostic score, age at diagnosis and tumor stages III and IV (compared to stage I) were significantly associated with overall survival (Figure 6C). These data confirmed that our prognostic score has clinical validity independent of tumor stage and age at diagnosis (p-value=0.007, HR=2.1, 95% CI:1.2-3.7) (Figure 6C).

**Figure 7:**
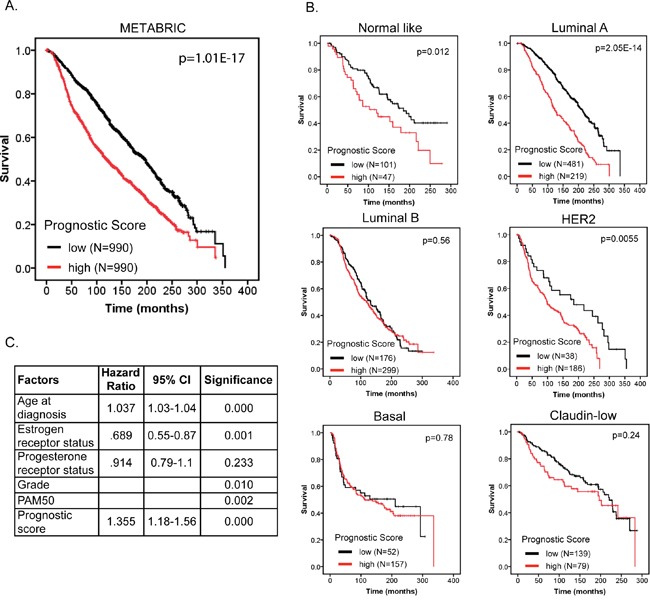
The 12-gene signature predicts overall survival independent of clinical factors and molecular subtypes **A**. Kaplan-Meier overall survival curve for breast cancer patients according to prognostic score using the 12-gene signature. The BC patient cohort was divided into two equal groups based on the prognostic score. The log-rank p-value of the curve comparison between the groups is shown. **B**. Kaplan-Meier overall survival curve for breast cancer patients stratified by molecular subtype. The log-rank p-value of the curve comparison between the groups is shown. **C**. The hazard ratio and the 95% confidence interval was calculated using a Cox model based on tumor grade, estrogen receptor and progesterone receptor status, age at diagnosis, molecular subtype (PAM50) and prognostic score as covariates.

We further validated our 12-gene prognostic signature in a second independent breast cancer dataset consisting of 1980 BC patients and containing data for individual breast cancer molecular subtypes (METABRIC; [17, 18]). Prognostic scores for all 1980 patients were calculated as described above for the TCGA cohort and patients were ranked based on their score and divided into two equal sized cohorts. Kaplan-Meier analysis revealed a significant difference between the two patient cohorts (Figure 7A; p=1.01E-17). To address the interaction of our signature with breast cancer molecular subtypes we stratified our patient cohort by molecular subtype (based on PAM50; [19]) and used Kaplan-Meier analysis to investigate differences in survival between the low and high prognostic score cohorts. We found that higher prognostic score was significantly associated with shorter survival in “normal-like”, “luminal A” and “HER2” subtypes (Figure 7B). To determine, in this data set, if our prognostic score was independent of age at diagnosis, tumor grade, estrogen- and progesterone-receptor status and molecular subtype (PAM50) we ran multivariate Cox regression force-entry with these factors including the prognostics scores as covariates. We further confirmed that our prognostic signature has clinical validity independent of age at diagnosis, estrogen receptor status, tumor grade and molecular subtype.

## DISCUSSION

Selecting patients who would most likely benefit from adjuvant systemic therapy is important considering the associated risks of treatment; the development of prognostic biomarkers is useful in this regard. While it remains difficult to identify good targets for the development of targeted therapies, cancer genome analysis has shown great promise in identifying key aberrations in tumor growth and survival pathways that could serve as prognostic biomarkers and targets for therapeutic intervention. We created a 12-gene prognostic scoring system, which robustly predicted BC patients’ RFS in independent breast cancer data sets. Our gene signature could guide adjuvant therapy for breast cancer patients and includes novel potential molecular targets for therapy. Genes in our signature did not overlap with existing gene signatures that predict breast cancer outcome and metastasis [20–22]. Multiple reasons can explain the lack of overlap between these signatures, including differences in sample size and data sets, clinical phenotypes and methods of signature development. Also, we have shown using co-expression network analysis that functionally related genes often strongly correlate in expression. Even though different signatures select different genes, they may still originate from co-expression cliques representing the same biological function. For example, the Oncotype DX gene signature, which is prognostic of breast cancer recurrence, consists of 16 cancer genes. Five of these genes were also included in our analysis (MKi67, STK15, BIRC5, CCNB1 and MMP11), but were not selected in our final gene signature. However, MKi67, STK15, BIRC5 and CCNB1 were all part of the same strongly interconnected and cell-cycle enriched co-expression clique. Our analysis selected NUF2, MELK and RNASEH2A from the same clique, however, given the strong correlations in expression, any one of the highly connected genes is likely to perform equally well. Using multivariate Cox regression with our 12-gene signature and the Oncotype DX 16-gene signature, we determined that our 12-gene signature was independent (p<0.005; HR=2.4, 95% CI:1.7-3.4), but equally important as the Oncotype DX gene signature (p<0.005; HR=2.2, 95% CI: 1.3-3.7). Another important variable associated with breast cancer survival is molecular subtype. Using a cohort of 1980 breast cancer patients with approximately 30 years of follow-up we determined that our signature could predict breast cancer patient survival for “normal-like”, “luminal-A” and “HER2” subtypes, but not “luminal-B”, “basal” and “claudin-low” subtypes. We should point out that in our analysis patients were stratified into two equally sized cohorts based on the median prognostic score and then further stratified by molecular subtype. This resulted in unequally sized cohorts for each subtype, which could potentially have confounded our analysis. To test this, we generated equally sized cohorts based on prognostic score for each individual subtype. We first stratified patients by molecular subtype and then further stratified patients inside each subtype by the median of the prognostic score. This analysis revealed similar observations as presented in Figure 7B confirming that our results are not confounded by unequally sized cohorts within different score groups. Future studies are granted to investigate whether our prognostic score can predict sensitivity to radiation- and/or chemotherapy.

The majority of the genes in our signature have previously been associated with cancer progression and patient outcome. MELK, NUF2 and ZBTB16 play important roles in cell cycle-related processes. Loss of ZBTB16 expression has been reported in a number of different tumor types including prostate cancer, non-small cell lung cancer, melanoma [23–25]. Overexpression of MELK, a serine/threonine kinase implicated in embryogenesis and cell cycle control has been identified in numerous human cancer types including breast, prostate, brain, colorectal and gastric cancer [26–30]. In BC, overexpression of MELK correlated with poor prognosis, whereas knockdown decreased proliferation [28, 30, 31]. NUF2 is part of a conserved protein complex associated with the centromere and plays a regulatory role in chromosomal segregation. Down regulation of NUF2 in pancreatic cancer cell lines inhibited tumor growth and enhanced apoptosis [32] whereas upregulation of NUF2 in colon cancer cells promoted tumorigenicity [33]. Overexpression of EPS15, which plays a role in terminating growth factor signaling, was shown to be a favorable prognostic factor in BC [34, 35]. Our signature also included the inner mitochondrial membrane protein TIMM17A. Decreased expression of TIMM17A reduced the aggressiveness of BC cells and TIMM17A expression was significantly associated with BC survival [36–38]. PLEKHH2 and TSC1 are involved in cell adhesion and actin dynamics. Loss of TSC1 was shown to result in the deregulation of cell motility and adhesion [39]. A polymorphic variant of TSC1 was associated with delayed age at diagnosis of ER-positive ductal carcinomas [40]. Also, TSC1, in coordination with TSC2, inhibits MTOR, which promotes cell growth and cell cycle progression [41]. PLPP1 degrades lysophosphatidate and is often down-regulated in tumor types. Using syngeneic and xenograft mouse models showed that PLPP1 overexpression in BC cells decreased tumor growth and the metastasis [42]. S100P is overexpressed in a variety of human tumor types [43]. S100P transcription is influenced by a number of signaling molecules including progesterone, androgens, glucocorticoids, BMP4 and IL6 and through interactions with a various proteins integrates and regulates multiple signaling pathways involved in degradation of extracellular matrix, invasion and metastasis and tumorigenesis (reviewed in [44]).

The role of PLEKHH2, USP47 and THYN1 has not been extensively studied in cancer progression. PLEKHH2 protein was enriched in renal glomerular podocytes, and shown to interact with focal adhesion proteins and actin to stabilize the actin cytoskeleton [45]. USP47 plays an important role in base-excision repair and the maintenance of genome integrity [46]. Depletion of USP47 induced accumulation of Cdc25A and decreased cell survival [47]. However, our results indicate that patients with high breast tumor expression of USP47 have significantly better relapse-free survival compared to patients with low breast tumor expression of USP47 (HR=0.65; p-value=2.40E-07). Thus, the exact role of USP47 in BC has yet to be determined. The role of THYN1 in BC is currently unknown, however, downregulation of THYN1 has been correlated with the induction of apoptosis in a specific B-cell lymphoma cell line [48].

Our gene co-expression network analysis identified a number of potential therapeutic targets. We found that 7 genes CREBRF, DIXDC1, AHNAK, CYBRD1, NOSTRIN, TNS2 and TNFSF12 were negatively correlated with the strongly interconnected cell cycle and mitosis clique. Indeed, a number of these genes have been identified as candidate tumor suppressor genes including CREBRF, DIXDC1, AHNAK and TNS2 [49–52]. Furthermore, NOSTRIN was found to be a potential negative regulator of disease aggressiveness in pancreatic cancer and CYBRD1 was identified as part of an iron regulatory gene signature that predicts outcome in BC [53, 54]. TNFSF12 (TWEAK) can promote cell death in tumor cell lines under certain conditions [55–57], and may also activate local macrophages to inhibit tumor progression [58]. The negative correlation of these 7 genes with the cell cycle enriched gene co-expression clique was observed in the co-expression network of breast tumor samples, but not the normal breast tissue co-expression network. This suggests that a therapeutic approach that increases expression of one or more of these 7 genes could collapse the tumor cell cycle machinery, while sparing adverse effects in healthy tissue.

In summary, we have generated a prognostic scoring system and 12-gene signature that is prognostic of BC patient relapse-free survival. Furthermore, using co-expression network analysis, we investigated the genetic architecture of RFS associated genes in normal and tumor tissues and identified 7 potential therapeutic targets that could be developed to target the tumor cell cycle machinery. Our analysis pipeline could furthermore be applied to other tumor types.

## MATERIALS AND METHODS

### Data sets used in this study

Gene transcript data of normal and tumor breast tissues was obtained from NCBI GEO accession numbers: GSE3744 (40 invasive ductal carcinoma samples and 7 normal breast samples), GSE10780 (42 invasive ductal carcinoma samples and 143 normal breast samples), GSE21422 (5 invasive ductal carcinoma samples and 5 normal breast samples) and GSE29044 (72 invasive ductal carcinoma samples and 36 normal breast samples). Normal breast gene transcript data used for generating gene expression correlation networks was obtained from GTEX (http://www.gtexportal.org/home/datasets) using the RPKM normalized gene transcript counts table [9, 10].

### Statistical analysis

GEO2R was used to calculate the differential expression of tumor versus normal using a fold-change cutoff of 1.5 and adjusted p-value 0.01. Association of differentially expressed genes and relapse-free survival in breast cancer patients was assessed using Kaplan- Meier plotter (http://kmplot.com) including KM survival analysis, hazard ratio with 95% confidence interval and logrank p-value for each gene using all available patients (not restricted to any clinical parameters such as grade, PR status, etc) [16].

Gene ontology enrichment analysis was performed using the web-based gene set analysis toolkit (adjusted p<0.05 was used as a threshold for significance) (http://bioinfo.vanderbilt.edu/webgestalt/) [59, 60].

### Gene co-expression network construction

Gene expression Spearman correlation coefficients were calculated in “R” for 795 probes (587 genes) that were differentially expressed between breast tumor and normal tissues samples. A gene network was generated where nodes represent individual genes and edges connecting nodes were drawn when the correlation coefficient exceeded |R|≥0.6 (adjusted p-value≤7.911E-08). The gene co-expression network was visualized using Cytoscape 3.1.1. (http://www.cytoscape.org).

### Prognostic gene signature

BC microarray data (GSE6532), describing RFS status and gene expression for our 357-gene panel, was collected for 393 patients. Sixty random samplings of 300 patients were extracted from this dataset and used as training sets to identify a biomarker panel associated with RFS. The residual 93 patients from each sample were used as test sets to validate the prognostic significance of the biomarker panel. A forward-conditional Cox regression using all 357 genes as covariates was performed using SPSS on each of the training sets in order to identify the biomarker panel. The results of each test were recorded and the genes that appeared in more than half of the training sets were included in our biomarker panel.

Cox regression was repeated on all 60 training sets using our 12-gene signature as covariates using the forced-entry (enter) method to obtain the beta values (coefficient) for each biomarker. The resulting 60 beta values of each biomarker were averaged to estimate the true beta value of each gene. A prognostic scoring system was created based on this formula:

$$\sum_{i=1}^{12}\left(\text{gene }i\;\beta \right)\;x\;(\text{gene }i\text{ expression level})$$

The patients were ranked by their prognostic score and divided into two equal sized cohorts. Kaplan-Meier plots were constructed and a long-rank test was used to determine differences among relapse free survival.

Prognostic scores for each of the test set samples were then calculated using the same set of mean beta values developed in the training set. Patients were ranked based on their prognostic score and divided into two cohorts based on the average prognostic-score cut-point in the training sets. Kaplan-Meier plots were constructed and a log-rank test was used to determine differences among RFS.

To further validate our biomarker panel, mRNA expression levels (normalized RNA-seq mRNA expression z-scores) for our 12-gene signature were obtained from cBioPortal for 1100 breast cancer samples (TCGA; http://www.cbioportal.org/data_sets.jsp) [61, 62] and for 1980 breast cancer samples (METABRIC) [17, 18]. New beta values for each of the twelve biomarkers were obtained using Cox regression. Prognostic scores were calculated and patients were ranked based on their score and divided into two equal sized cohorts. Kaplan-Meier analysis and a log-rank test were used to determine differences in survival.

## SUPPLEMENTARY TABLES










