# Supplementary material for: Genome-wide screen identifies a novel prognostic signature for breast cancer survival
Source: Oncotarget. 2017 Jan 21;8(8):14003–16. doi: 10.18632/oncotarget.14776 (PMC5355157; doi:10.18632/oncotarget.14776)
Supplement: Supplementary file 1 [file oncotarget-08-14003-s001.pdf]

Genome-wide screen identifies a novel prognostic signature for breast cancer survival

SUPPLEMENTARY TABLES

Supplementary Table 1: Differential gene expression between breast tumor and normal breast tissue

See Supplementary File 1

Supplementary Table 2: The 795 probe IDs robustly deregulated in breast cancer

See Supplementary File 2

Supplementary Table 3: Genes significantly associated with relapse-free survival in breast cancer patients

See Supplementary File 3

Supplementary Table 4: Significant GO categories associated with 381 genes significantly associated with relapse-free survival in breast cancer

See Supplementary File 4
